# Meta-analysis of the effects of denosumab and romosozumab on bone mineral density and turnover markers in patients with osteoporosis

**DOI:** 10.3389/fendo.2023.1188969

**Published:** 2023-07-12

**Authors:** Mingwei Hu, Yifan Zhang, Jianjun Guo, Cuicui Guo, Xue Yang, Xue Ma, Hao Xu, Shuai Xiang

**Affiliations:** Department of Joint Surgery, The Affiliated Hospital of Qingdao University, Qingdao, China

**Keywords:** osteoporosis, denosumab, romosozumab, bone mineral density, bone turnover marker

## Abstract

**Purpose:**

To assess the alterations in bone mineral density and bone turnover marker concentrations following the administration of denosumab and romosozumab therapies in patients with osteoporosis.

**Methods:**

PubMed was searched for studies published until January 28, 2023, that investigated the clinical efficacy and bone turnover marker changes of denosumab and romosozumab in the treatment of osteoporosis, with a minimum follow-up of 3 months in each study. Studies were screened, and data on changes in bone mineral density (BMD), P1NP, and TRACP-5b levels after treatment were extracted and included in the analysis.

**Results:**

Six studies were analyzed. At 3 months after treatment, the romosozumab group showed greater changes in lumbar BMD and bone turnover markers. BMD of total hip and femoral neck was relatively delayed. Beginning at 6 to 12 months, romosozumab showed greater changes in bone mineral density and markers of bone turnover.

**Conclusion:**

Both romosozumab and denosumab have antiosteoporotic effects, with greater effects on BMD and bone turnover markers observed within 12 months of romosozumab treatment.

**Systematic Review Registration:**

https://www.crd.york.ac.uk/prospero, identifier CRD42023395034.

## Introduction

Population aging is becoming an increasingly prominent global problem. With the increase in the number of older adults, osteoporosis has become a great challenge. Osteoporosis is a disease characterized by low bone mass and destruction of the bone structure, resulting in impaired bone strength and increased fracture risk, often without symptoms until the first fracture occurs (1). However, fractures in older adults are often catastrophic, and femoral neck fractures are even called “the last fracture.” Some studies have pointed out that falls in older adults may be the result of fractures rather than the cause (2). Fragility fractures caused by osteoporosis significantly increase the risk of fractures. Therefore, controlling the occurrence and progression of osteoporosis in the older population has become a concern.

Denosumab and romosozumab have received increasing attention as monoclonal antibodies with antiosteoporotic effects. Among these, denosumab has been widely used in clinical practice and has achieved significant results in the treatment of patients with senile osteoporosis and bone tumors. Denosumab is an abundant human monoclonal antibody that binds to the receptor activator of nuclear factor kappa-B (RANK) ligand (RANKL) on osteoclasts (3, 4), thereby inhibiting bone resorption. It is one of the most widely used antiresorptive drugs in clinical practice (5). Romosozumab is a monoclonal antibody that binds to and inhibits sclerostin with the dual efficacy of increasing bone formation and decreasing bone resorption (6, 7). Sclerostin is secreted by osteocytes, negatively regulates osteoblast-mediated bone formation, and antagonizes Wnt signaling by binding to low-density lipoprotein receptor-associated protein 5/6 (LRP5/6) (8–14). Both affect osteogenesis and osteoclasts through different mechanisms and play a role in the treatment of osteoporosis. However, there are no relevant evidence-based medical studies comparing the clinical effects of these two drugs in the treatment of osteoporosis. We hope to analyze the existing controlled studies to clarify the clinical effect of the two drugs in the treatment of osteoporosis and the changes in bone metabolism markers and to provide guidance for subsequent clinical application and selection.

## Materials and methods

This meta-analysis was performed in accordance with the Preferred Reporting Items for Systematic Reviews and Meta-Analyses (PRISMA) statement and registered with PROSPERO (CRD42023395034).

### Search strategy

PubMed was used to examine the effects of denosomab and romosozumab on bone mineral density (BMD) and bone metabolism in patients with osteoporosis. Searches were performed using the terms “(Denosumab) and (Romosozumab),” and no language restrictions were applied. Articles published between January 2011 and January 2023 were selected. The final search was conducted on January 28, 2023.

### Article selection process

**Figure 1:**
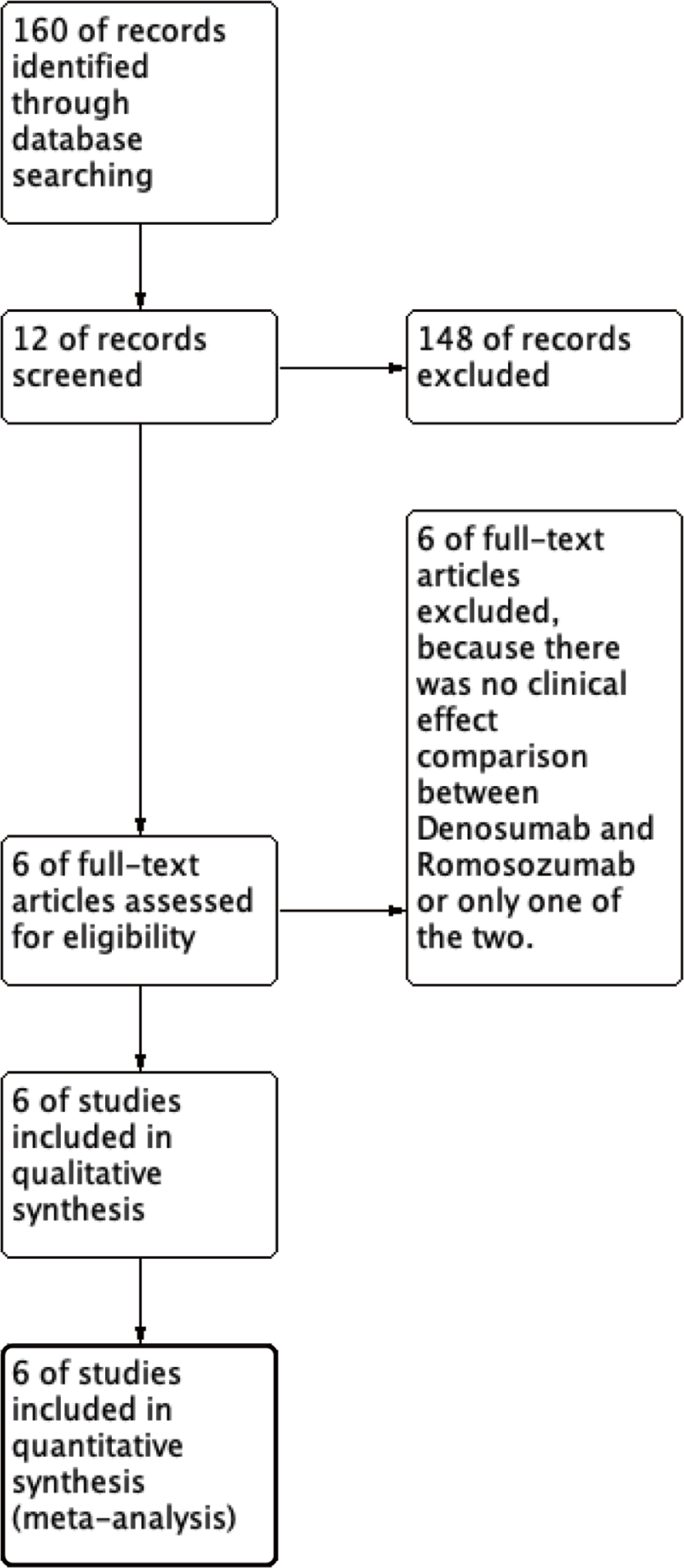
Flow diagram of the study.

We are currently enrolling patients with osteoporosis who have received denosumab or romosozumab. Two treatment groups, denosumab, and romosozumab, were compared. The outcomes were changes in BMD, P1NP, and TRACP-5b levels with denosumab or romosozumab treatment. The study types were retrievable, retrospective, randomized controlled, and cohort studies. The primary screening was performed as follows: We included all article types, including the terms “Denosumab and Romosozumab,” and links to the full text of articles are available on the Internet and from search sites. In the secondary screening, studies that did not present accurate mean data and those that were not computable using graphs were eliminated because complete data were not available (**Figure 1**).

### Quality assessment

Two authors independently checked and selected all the references. When the results were inconsistent, a third party provided an opinion to resolve the issue.

The Cochrane Collaboration Risk of Bias Tool (15) was used to evaluate the quality of selected studies. Funnel plot asymmetry was used to assess the publication bias.

### Data extraction

Data were extracted from all studies included in this analysis (author, year of publication, number of patients, and BMD [lumbar spine, total hip, or femoral neck], P1NP, and TRACP-5b). To evaluate the rates of change in BMD, P1NP, and TRACP-5b in patients with osteoporosis treated with denosumab and romosozumab when the original database was not extractable, we manually calculated these values using the information available in published charts. In studies reporting only median and quartile ranges or multiple ranges, means and standard deviations were calculated using the method described by Wan et al. (16).

### Data synthesis

A meta-analysis was performed to evaluate the efficacy of denosumab and romosozumab for the treatment of osteoporosis. Clinical data before and after treatment were analyzed. Outcomes are expressed as means or mean differences with 95% confidence intervals using random effects models. Heterogeneity was assessed using the I^2^ test, in which I^2^ values of 25%, 50%, and 75% were defined as low, moderate, and high, respectively (17). All analyses were performed using Review Manager (Revman) 5.4.

## Results

### Study selection

This study identified 12 records from 158 studies in PubMed, of which six were removed through initial screening. The final six studies (18–23) met the selection criteria and were included in the meta-analysis. In a study conducted by Shimizu et al. (20), vitamin D or bisphosphonate (BPs) intervention was administered after denosumab or romosozumab, and the patients were grouped according to the intervention.

### Characteristics of the studies and patient background

A total of 247 patients who received denosumab and 224 who received romosozumab were enrolled in this study. A summary of the baseline characteristics of the patients in each study is provided in **Table 1**.

**Table 1:** Characteristics of included studies in this meta-analysis.

Authors(Year)	Level evidence	Group	Number of subjects	Age (year), mean (s.d)	Female (%)	BMI (Kg/m^2^), mean (s.d)	T-score (lumbar spine), mean (s.d)	T-score (total hip), mean (s.d)	T-score (femoral neck), mean (s.d)	P1NP (μg/L), mean (s.d)	TRACP-5b (mU/dl), mean (s.d)	
Kobayakawa 2022	Level III	Denosumab	36	70.0 (11.2)	31 (86.1)	21.3 (3.7)	-1.79 (1.42)	-2.33 (0.88)	-2.53 (0.82)	34.9 (21.0)	378.2 (225.4)	
		Romosozumab	36	71.2 (13.3)	30 (82.9)	20.4 (3.4)	-1.79 (1.44)	-2.71 (1.08)	-2.39 (1.15)	44.5 (38.0)	296.5 (143.6)	
Kobayakawa 2021	Level III	Denosumab	69	74.20 (11.32)	Not mentioned	21.15 (3.39)	-2.50 (1.13)	-2.55 (0.73)	-3.12 (0.62)	59.1 (36.6)	476.6 (205.2)	
		Romosozumab	69	75.83 (9.70)	Not mentioned	22.09 (3.24)	-2.62 (1.25)	-2.57 (0.84)	-3.12 (0.82)	70.0 (44.3)	528.4 (255.2)	
Mochizuki 2022	Level III	Denosumab	26	74.8 (7.9)	Not mentioned	21.1 (2.6)	-2.1 (1.1)	-2.4 (1.0)	-2.9 (0.7)	47.6 (29.0)	455.7 (169.8)	
		Romosozumab	25	72.0 (7.7)	Not mentioned	21.7 (3.2)	-1.8 (1.0)	-2.1 (0.5)	-2.6 (0.6)	35.6 (16.0)	388.3 (164.7)	
Shimizu 2021 (Vit D)	Level III	Denosumab	38	75.3 (5.4)	Not mentioned	22.1 (2.6)	Not mentioned	Not mentioned	Not mentioned	62.7 (26.5)	497.6 (158.8)	
		Romosozumab	43	71.8 (6.3)	Not mentioned	22.7 (2.1)	Not mentioned	Not mentioned	Not mentioned	56.3 (24.3)	451.0 (116.1)	
Shimizu 2021 (BPs)	Level III	Denosumab	35	73.7 (7.3)	Not mentioned	20.0 (2.6)	Not mentioned	Not mentioned	Not mentioned	30.2 (19.6)	285.5 (121.6)	
		Romosozumab	38	74.4 (6.4)	Not mentioned	22.2 (2.6)	Not mentioned	Not mentioned	Not mentioned	21.6 (8.5)	279.5 (86.2)	
Jeong 2021	Level III	Denosumab	21	66.0 (8.6)	Not mentioned	22.9 (2.5)	-2.40 (0.73)	Not mentioned	Not mentioned	Not mentioned	Not mentioned
		Romosozumab	10	66.8 (8.1)	Not mentioned	23.1 (3.8)	-2.31 (0.41)	Not mentioned	Not mentioned	Not mentioned	Not mentioned
Mochizuki 2022	Level III	Denosumab	25	72.6 (7.1)	Not mentioned	Not mentioned	-1.8 (1.1)	-2.1 (0.4)	-2.6 (0.5)	34.3 (20.0)	360.1 (147.8)	
		Romosozumab	25	74.7 (7.9)	Not mentioned	Not mentioned	-2.0 (1.4)	-2.0 (1.4)	-3.0 (0.7)	41.4 (24.6)	427.2 (177.7)	

### Effect of denosumab and romosozumab treatment on BMD and bone turnover markers at 3 months

**Figure 2:**
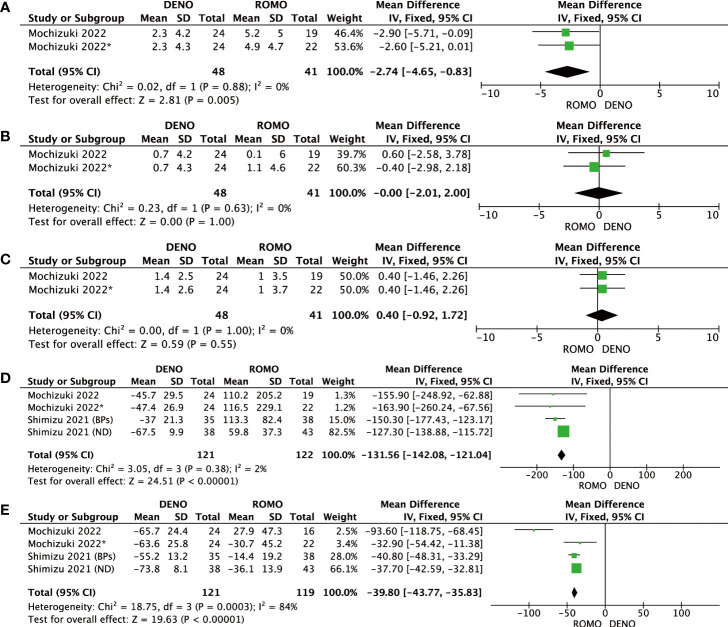
**(A)** Mean difference of BMD in the lumbar spine at 3 months from baseline between denosumab and romosozumab. **(B)** Mean difference of BMD in the femoral neck at 3 months from baseline between denosumab and romosozumab. **(C)** Mean difference of BMD in the total hip at 3 months from baseline between denosumab and romosozumab. **(D)** Mean difference of P1NP at 3 months from baseline between denosumab and romosozumab. **(E)** Mean difference of TRACP-5b at 3 months from baseline between denosumab and romosozumab. BMD, bone mineral density; DENO, denosumab; ROMO, romosozumab.

BMD at the lumbar spine, femoral neck, total hip, P1NP, and TRACP-5b were evaluated 3 months after denosumab and romosozumab treatment. BMD in the lumbar spine (P=0.005, I2 = 0%), P1NP (P< 0.00001, I2 = 2%), and TRACP-5b (P<0.00001, I^2 ^= 84%) showed significant differences at 3 months, whereas BMD in the femoral neck (P=0.63, I2 = 0%) and total hip (P=1.00, I2 = 0%) showed no significant difference. Based on these results, after 3 months of treatment with romosozumab, bone metabolic markers showed greater clinical relevance than with denosumab. In contrast, BMD was significantly different only in the lumbar spine and not in the femoral neck or total hip (**Figure 2**).

### Effect of denosumab and romosozumab treatment on BMD and bone turnover markers at 6 and 12 months

**Figure 3:**
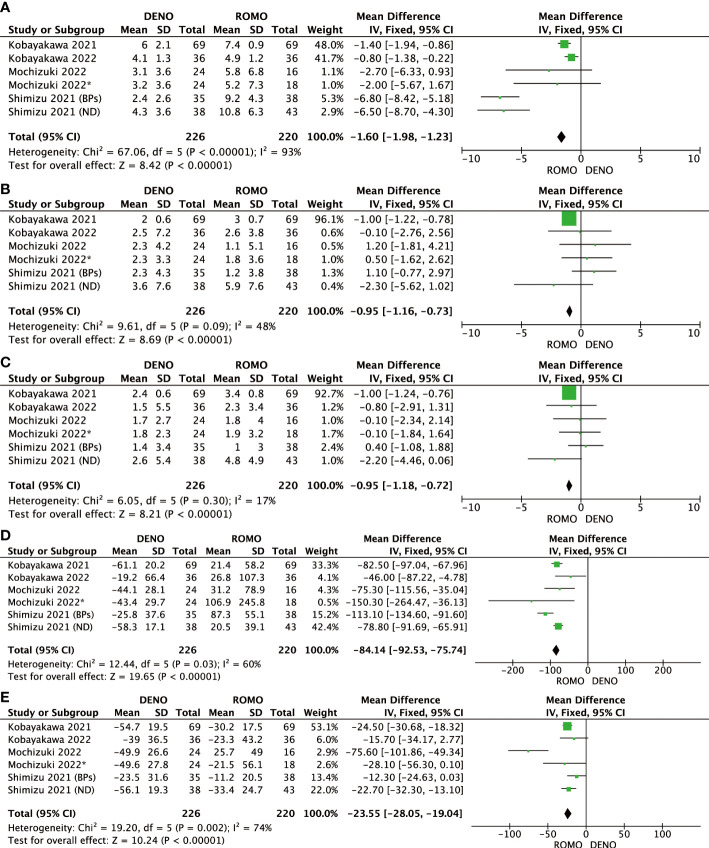
**(A)** Mean difference of BMD in the lumbar spine at 6 months from baseline between denosumab and romosozumab. **(B)** Mean difference of BMD in the femoral neck at 6 months from baseline between denosumab and romosozumab. **(C)** Mean difference of BMD in the total hip at 6 months from baseline between denosumab and romosozumab. **(D)** Mean difference of P1NP at 6 months from baseline between denosumab and romosozumab. **(E)** Mean difference of TRACP-5b at 6 months from baseline between denosumab and romosozumab. BMD, bone mineral density; DENO, denosumab; ROMO, romosozumab.

**Figure 4:**
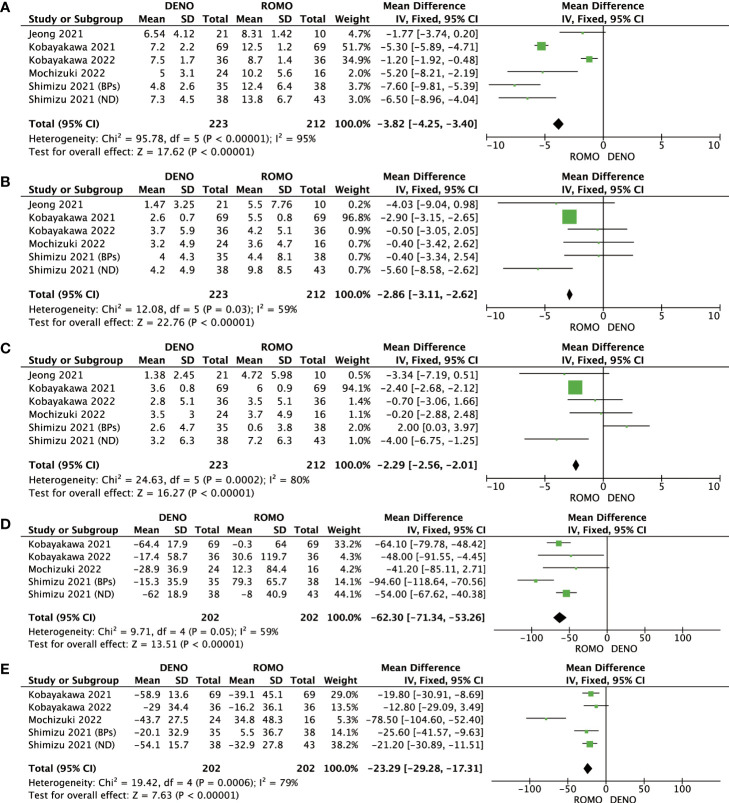
**(A)** Mean difference of BMD in the lumbar spine at 12 months from baseline between denosumab and romosozumab. **(B)** Mean difference of BMD in the femoral neck at 12 months from baseline between denosumab and romosozumab. **(C)** Mean difference of BMD in the total hip at 12 months from baseline between denosumab and romosozumab. **(D)** Mean difference of P1NP at 12 months from baseline between denosumab and romosozumab. **(E)** Mean difference of TRACP-5b at 12 months from baseline between denosumab and romosozumab. BMD, bone mineral density; DENO, denosumab; ROMO, romosozumab.

Romosozumab showed superior clinical efficacy to denosumab in terms of BMD of the lumbar spine, femoral neck, total hip, P1NP, and TRACP-5b after 6 and 12 months of treatment. All these differences were significant (P<0.00001). Based on this analysis, romosozumab showed superior clinical outcomes compared to denosumab at 6 months after treatment (**Figures 3**, **4**).

## Discussion

Osteoporosis is a common challenge in older adults and has received extensive attention. Denosumab and romosozumab are clinically useful in the field of orthopedics or endocrine therapy. Therefore, we conducted this study, which showed the superior effect of romosozumab on BMD of the lumbar spine and bone turnover markers, with significant differences. However, there was no significant difference in the BMD between the femoral neck and total hip at 3 months. Significant differences in all measures were observed after 6 months of treatment and were sustained at 12 months, with greater antiosteoporotic effects observed with romosozumab.

Denosumab, a fully human monoclonal antibody, blocks RANK activity by binding to RANKL, an osteoproteger that acts as a nuclear factor kappa-B ligand (RANKL) receptor agonist to regulate bone resorption and prevent RANK receptor activation in osteoclasts and precursor cells. However, it has a longer half-life and more effective antiresorptive activity than osteoprotegerin (24). Osteocytes also release sclerostin, which antagonizes the Wnt signaling pathway, leading to bone resorption (25). Sclerostin, encoded by the SOST gene and released by osteocytes, binds to LRP5/6 to block sites normally occupied by Wnt signaling pathways and induces bone resorption (26–28).

The 2022 UK Clinical Guidelines for the Prevention and Treatment of Osteoporosis (29) state that the delivery of oral bisphosphonates or intravenous zoledronate is the most cost-effective intervention, with alternative options such as denosumab, hormone replacement therapy, or raloxifene. In particular, a long-term anti-osteoporosis management plan should be in place prior to the use of denosumab; denosumab treatment should not be discontinued or delayed to avoid unintended discontinuation, which can lead to an increased risk of vertebral fracture. However, this indicates that it can be used for a long time when alternative therapies are not considered.

The current study reveals that romosozumab exhibits advantageous effects on lumbar bone turnover markers and bone mineral density following a 3-month period of treatment. Nonetheless, for BMD changes at the femoral neck and total hip, there is a relatively lagged effect that does not differ significantly. Our findings align with a study conducted by McClung et al. (30), which could be attributed to the biological and structural differences between spinal and hip bones. The spinal skeleton is more metabolically active than the hip bone, suggesting a greater rate of bone formation and resorption. Furthermore, the spinal bone presents a larger surface area, which enhances the detection of more significant BMD changes in a shorter period. Conversely, the hip bone is denser and presents a smaller surface area compared to the spine, leading to a slower manifestation of BMD changes that take longer to detect. Based on the results of our study, a possible clinical significance could be proper monitoring of BMD changes in osteoporosis patients to assess treatment efficacy. The different rates of BMD changes occurring at various sites highlight the need for multi-site monitoring of BMD to enhance the understanding of the overall effectiveness of osteoporosis treatment.

Romosozumab has a dual effect on bone metabolism, stimulating bone formation and inhibiting bone resorption. Several studies have shown that it is beneficial for bone density recovery (7, 30, 31) and has a low risk of fracture (32). Chavassieux et al. (31). performed an iliac bone biopsy through a Fracture Study in Postmenopausal Women with Osteoporosis (FRAME). Micro-computed tomography (µCT) and histological analyses revealed that romosozumab contributed to increased bone mass, bone volume (BV/TV), cortical thickness (Ct.Th), and trabecular thickness (Tb.Th). In addition, CT analysis showed that it could improve trabecular connectivity, significantly reduce trabecular bone pattern factor (TBPf), and increase trabecular tissue BMD (Tb.TMD). This indicates that romosozumab can significantly improve bone metabolism at the level of the bone tissue structure. The anti-reabsorption effect of romosozumab may be achieved through the Wnt pathway-mediated upregulation of osteoprotectin (OPG). A murine IgG1 sclerostin-neutralizing monoclonal antibody (Scl-AbII) reversed the mineralizing inhibition of sclerostin binding in mouse MC3T3-E1 osteoblasts (33). Serum metabolic markers indicated a complex pattern of bone formation after romosozumab treatment, with an early increase and late decline (7, 32, 34). This may explain the changing trends of P1NP and TRACP-5b levels in the romosozumab group in this study. Long-term administration could reduce the P1NP and TRACP-5b levels to baseline levels.

Regarding safety, the primary outcome and safety and tolerability results for denosumab were satisfactory in the 7–10 years of the FREEDOM trials (35). A total of 13 cases of osteonecrosis of the jaw were observed during follow-up, of which 8 cases occurred during the first 5 years of the extended study, and the other 5 cases were observed during 8–10 years. This suggests a possible association between the risk of jaw osteonecrosis and the duration of denosumab treatment (36). Romosozumab demonstrated superior resistance to osteoporosis and fracture risk, with similar rates of adverse and severe adverse events during double-blindness in the FRAME (32), ARCH (37), and BRIDGE (38) trials. However, reports of vascular risk at the center of the trial have attracted attention, mainly reporting the occurrence of major cardiovascular adverse events, cardiovascular death, or myocardial infarction. Fixen et al. (39) suggested that this may be related to the non-bone expression of sclerosins. The expression of sclerosin in the vascular smooth muscle and valvular tissue may increase with increased calcification (40, 41), suggesting that sclerosin negatively regulates vascular calcification, which may be associated with an increased risk of cardiovascular complications associated with romosozumab use. The use of romosozumab in patients with osteoporosis and cardiovascular disease should be considered for cardiovascular complications or patient screening at the time of treatment initiation.

In this study, our findings suggest that denosumab or romosozumab treatments induce an increase in bone mineral density (BMD). Based on a meta-analysis of 38 studies conducted by Bouxsein et al., a greater reduction in fracture risk was associated with a significant improvement in BMD measured through dual-energy X-ray absorptiometry (DXA) (42). Despite the study’s limitations in determining the structural or biomechanical relationship between increased BMD and decreased fracture risk or anti-osteoporosis effects; or the relationship between BMD and the latter effects, our results contribute to the possibility of future research in the field of anti-osteoporosis.

In this study, a comprehensive analysis and evaluation of denosumab and romosozumab in patients with osteoporosis were conducted. However, it should be noted that the studies included in this review were recent studies, with up to 12 months of intervention, and no long-term follow-up or long-term studies assessed clinical outcomes. This may limit the inclusion of data in the analysis and prevent the drawing of long-term clinical conclusions. This may be related to the requirement for romosozumab for up to 12 months, followed by continued treatment with denosumab or bisphosphonates. Another limitation of this study is that too few subjects were included. Currently, there are no clinical studies with sufficient sample sizes to conduct controlled trials of denosumab and romosozumab. We believe that further studies with larger sample sizes and longer follow-up periods should be conducted for clinical treatment and large-sample evidence-based meta-analyses should be encouraged.

## Conclusion

Denosumab and romosozumab have favorable effects on osteoporosis. After 3 months of treatment, romosozumab showed an advantage in bone turnover markers and BMD in the lumbar spine. In contrast, changes in BMD in the femoral neck and total hip were relatively delayed, and no significant differences were observed. From 6 to 12 months after treatment, romosozumab performed significantly better than denosumab in all observed measures.

## Data availability statement

The original contributions presented in the study are included in the article/**Supplementary Material**. Further inquiries can be directed to the corresponding authors.

## Author contributions

MH: Methodology, Validation, Formal analysis, Investigation, Data curation, Writing- Origin Draft; Visualization; YZ: Methodology, Resources; JG: Formal analysis, Investigation, Data curation; CG: Investigation; Visualization; XY: Resources, Data curation; XM: Resources, Data curation; HX: Writing- Review & Editing; Supervision SX: Conceptualization; Methodology; Writing- Review & Editing; Supervision; Project acquisition. All authors contributed to the article and approved the submitted version.
