# Supplementary figures and images for: Meta-analysis of the effects of denosumab and romosozumab on bone mineral density and turnover markers in patients with osteoporosis
Source: Front Endocrinol (Lausanne). 2023 Jul 12;14:1188969. doi: 10.3389/fendo.2023.1188969 (PMC10390296; doi:10.3389/fendo.2023.1188969)

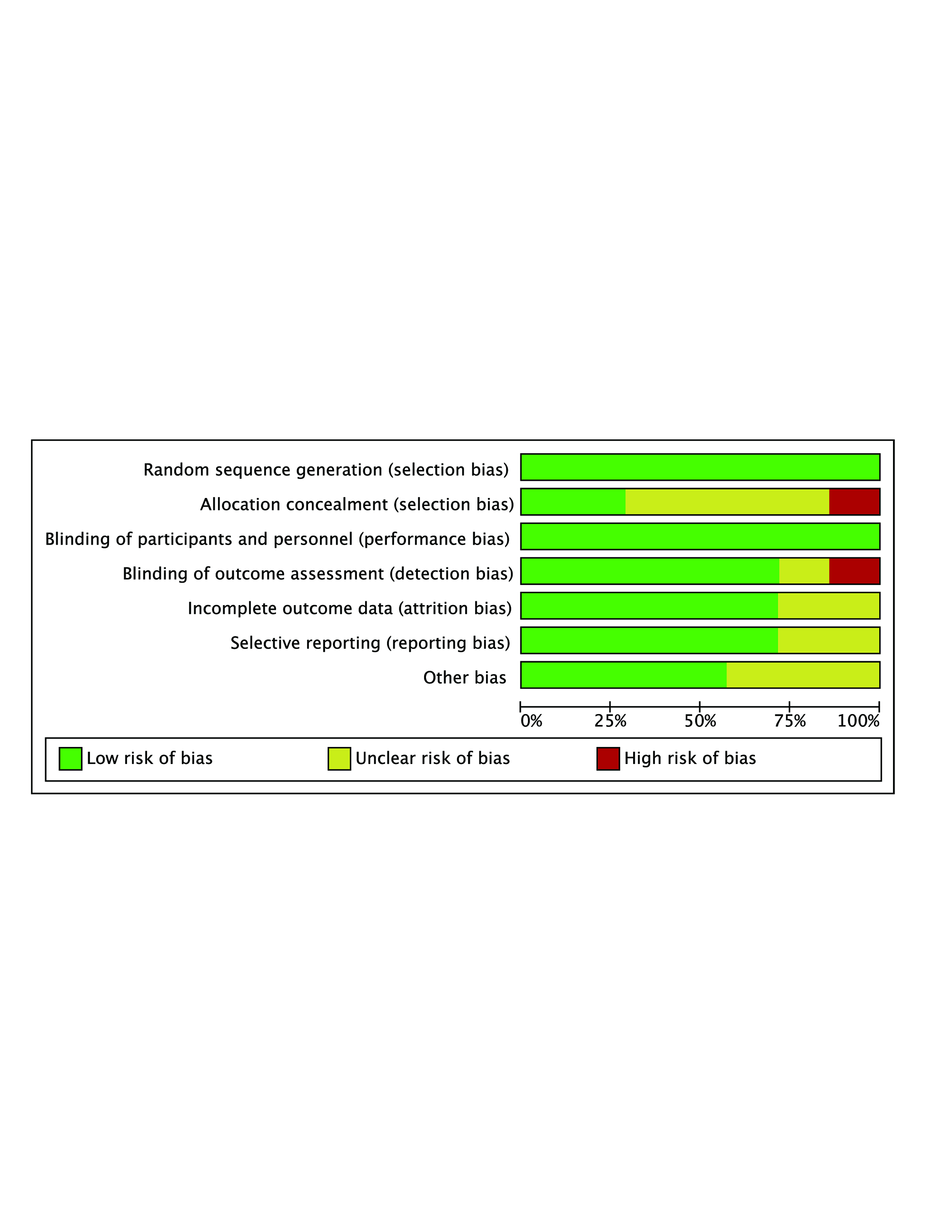

Supplement: Supplementary file 1 [file Image_1.tif]

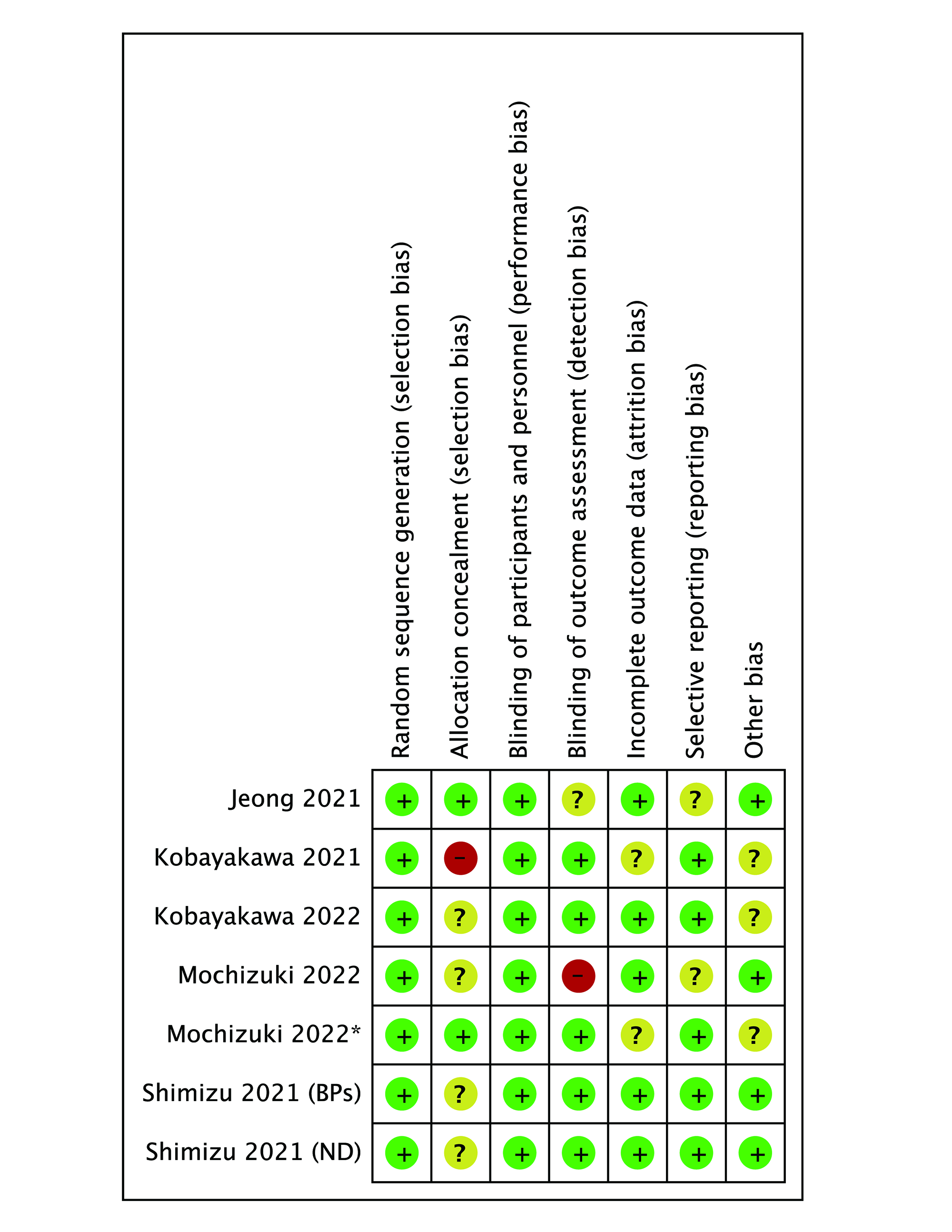

Supplement: Supplementary file 2 [file Image_2.tif]
